# An analysis of the stress levels, influencing factors and countermeasures of kidney transplant recipients in a certain hospital

**DOI:** 10.3389/fpubh.2025.1690720

**Published:** 2025-10-20

**Authors:** Jingyi Jiang, Lin Wu, Leiqun Xiong, Bin Zhao, Shaoping Tian, Houzhao Wang, Xiaoying Lv

**Affiliations:** 1 Department of Transfusion, Zhongshan Hospital (Xiamen), Fudan University, Xiamen Clinical Research Center for Cancer Therapy, Xiamen, Fujian, China; 2 Department of Clinical Laboratory, Xiang’an Hospital of Xiamen University, School of Public Health, Xiamen University, Xiamen, Fujian, China

**Keywords:** kidney transplantation, recipient, stress, influencing factors, countermeasures

## Abstract

**Background:**

Kidney transplantation remains the most effective therapeutic intervention, significantly enhancing long-term survival rates and overall well-being. Patients undergoing kidney transplantation face challenges in terms of their physical, psychological and social adaptation, such as anxiety, depression and psychological stress resulting from changes in their lifestyle. Existing studies with insufficient analysis of psychological and social factors and systematic intervention strategies.

**Objective:**

This study aims to evaluate the current stress levels status of patients after kidney transplantation. Analyze influencing factors, and explore targeted intervention strategies, with the goal of informing comprehensive patient management and improving long-term outcomes.

**Methods:**

A cross-sectional study was conducted, enrolling recipients who underwent kidney transplantation at a tertiary-level hospital in Fujian Province between June 2019 and December 2024. A total of 202 valid questionnaires were collected. Demographic characteristics, disease-related information, and current stress levels were collected through questionnaire surveys. Independent samples t-tests or one-way ANOVA were performed on total stress scores. Variables with *p* < 0.05 were included in multiple linear regression analysis to assess the combined effects of independent variables on total stress scores.

**Results:**

A total of 202 samples were included. The patients’ total stress score was 57.78 ± 12.73, with an average score of 2.41 ± 0.53 per item. Kidney transplant recipients generally experience moderate stress levels, with financial situation having the highest average score (2.91 ± 1.07), while the mean score for romantic and marital psychology is the lowest (1.86 ± 1.00). The economic situation of the family, factors related to the disease and knowledge of postoperative recovery are important factors that influence the level of stress experienced by patients (*p* < 0.05), accounting for 48.5% of the variance in stress levels.

**Conclusion:**

Kidney transplant recipients exhibited moderate levels of stress, with economic circumstances representing a predominant source of stress. The economic status of the patient’s family, factors associated with the illness, and knowledge of postoperative recovery had a significant impact on the overall stress levels. To alleviate stress and enhance the quality of life for these patients, it is recommended that financial assistance be provided, enhanced post-transplant management and educational programmes be implemented to promote knowledge about the condition.

## 1 Introduction

Chronic Kidney Disease (CKD) represents a significant public health challenge, affecting over 10% of the global population, with a higher prevalence in low- and middle-income countries (1). In 2017, the worldwide prevalence of CKD reached approximately 690 million individuals, contributing to an estimated 1.2 million deaths annually. Additionally, around 1.4 million cardiovascular mortality cases can be concurrently attributed to kidney pathology (2). Current research indicates that CKD patients commonly experience depression and a decline in quality of life (3, 4), however, studies focusing on the psychosocial stress dynamics and post-transplant psychological adaptation remain limited.

In the preceding decade, there has been a considerable advancement in the domains of education, healthcare services and environmental protection within China. This progression has culminated in a 30% decline in the prevalence of CKD within the Chinese population (5). However, with the ongoing aging of the Chinese population, the prevalence of CKD is expected to increase in the future. The risk of progression to End-stage kidney disease (ESRD) among CKD patients is also rising, posing significant challenges to the healthcare system and exerting substantial socio-economic pressures. ESRD represents the final phase of CKD, kidney transplantation is the most effective treatment for ESRD. Evidence indicates that transplant recipients exhibit significantly improved long-term survival rates and quality of life compared to those maintained on dialysis (6). It is important to note that the process is both complicated and protracted. It may encompass a number of stages, including the diagnosis of the illness, waiting for a suitable organ, surgery, and post-operative management. It is also important to recognize that all of these stages have the potential to place patients under considerable psychological strain (7).

After kidney transplantation, recipients may experience multiple stresses. Recipients need lifelong immunosuppressive therapy to prevent allograft rejection, which not only increases the risk of infection (8), but also may cause metabolic disorders, including new-onset diabetes (9), cardiovascular events (10) and cognitive decline (11). Long-term medication use can have an impact on patients’ physical and mental health, as well as placing a significant economic burden. Furthermore, changes in self-image and the difficulty of rebuilding social roles can further increase psychological pressure (12, 13). Although the 5-year and 10-year survival rates of transplanted kidneys are 76 and 53%, respectively, (14), recipients still face the risk of postoperative complications, including hypertension (15–17), infections (18, 19), and diabetes mellitus (20). Not only does long-term use of immunosuppressive treatment increases the risk of infection (18), it may also lead to poor transplant outcomes if recipients do not adhere to the treatment (21). Research has found that recipients aged 18 to 30 experience more gastrointestinal symptoms, and this is positively correlated with the adherence to immunosuppressive treatment (22). At the same time, quality of life improves with better sleep quality (23), and post-traumatic growth (PTG) can reduce depression (24). However, recipients of kidney transplants are at high risk of depression, anxiety and stress, which is closely related to the adherence to immunosuppressive drugs and their side effects (25).

Stress is a physiological and psychological response of the body to external stimuli. Prolonged stress can lead to immune dysregulation and endocrine imbalances, subsequently affecting disease prognosis. Research has found that recipients generally experience anxiety and depression (26), fear of disease recurrence, concern about drug side effects and changes in lifestyle can all lead to additional stress (27–29). Therefore, by evaluating the stress level of recipients can provide a more comprehensive understanding of the stress experienced by patients after kidney transplantation, and can inform the optimisation of patient care and improve long-term outcomes.

## 2 Materials and methods

### 2.1 Research design and participants

This observational study adopted a cross-sectional survey, with the research subjects being patients who underwent kidney transplantation in a tertiary hospital from June 2019 to December 2024.

### 2.2 Inclusion criteria

1. Being age 18 years or above.
2. Having ability to answer questions independently.
3. Agreeing to participate in the research.

### 2.3 Exclusion criteria

1. Age under 18 years.
2. Suffering from severe physical or mental illness.
3. Experiencing other significant stressful events within the past 6 months.

### 2.4 Data collection

The observational study obtained the consent of the sampling hospitals and departments to this study. Research team members received uniform training to ensure the consistency and accuracy of the data collection. The questionnaire was distributed and collected through a combination of online and offline methods. Staff members explained the purpose and significance of the study patients who met the inclusion criteria, obtained informed consent, and conducted the questionnaire survey. The study was conducted anonymously.

### 2.5 Participant characteristics form

The study was designed based on the research content and is divided into two parts: general information and disease-related situations. General demographic characteristics include gender, age, marital status, number of children, income, educational level, and occupation; disease-related situations include whether the primary disease is clear, the type of dialysis used before transplantation, the duration of dialysis, the waiting time for transplantation, the recovery of creatinine levels, and whether complications have occurred.

### 2.6 Survey on the current stress levels in kidney transplant recipients

Based on the Kidney Disease Quality of Life Instrument (30) (KDQOL-SF™1.3), the scale was adapted to reflect the health-related status of kidney transplant recipients. The questionnaire contains 24 items, covering five dimensions: health impact, romantic relationships, economic situation, living restrictions and support systems. It uses a 5-point Likert scale, ranging from “no impact (never)” to “severe impact (always),” with scores ranging from 1 to 5. Higher scores indicate greater patient stress. The questionnaire’s overall Cronbach’s alpha coefficient is 0.871, indicates good reliability The KMO value is 0.858 and Bartlett’s sphericity test *p* < 0.001. Factor analysis showed that the extracted factors cumulatively explained 64.293% of the total variance, indicating good construct validity. In our study, we categorized participants into low-pressure (scoring in the top 30%), medium-pressure (scoring between 30 and 70%), and high-pressure (scoring between 70 and 100%) groups.

### 2.7 Theoretical foundations

The selection of key variables in this study is grounded in the Stress and Coping Theory (31) and the Chronic Disease Self-Management Model (32). The Stress and Coping Theory emphasizes that stress is not solely determined by external events but is also significantly influenced by individuals’ subjective appraisals of these events and their coping strategies. The Chronic Disease Self-Management Model highlights that patients utilize self-management skills and resources to cope with the pressures imposed by chronic illnesses. Stressors are characterized as “negative factors” that deplete personal energy during the disease process. Conversely, resources are considered “positive factors” In kidney transplant recipients, stressors encompass health impacts, economic challenges, and lifestyle limitations (33, 34), while resources include social support, psychological resilience, and medical resources (13, 35). These stressors and resources directly influence patients’ mental health and quality of life. An imbalance between stressors and resources may contribute to increased disease-related stress, whereas resources serve a buffering role, mitigating the adverse effects of stressors on stress development.

### 2.8 Statistical analysis

The Statistical analysis was performed with IBM Statistical Package for the Social Sciences (SPSS) Statistics 26.0. First, descriptive statistical analysis was performed on the sample’s sociodemographic characteristics, disease-related situations, and stress levels, using frequency and percentage statistics. According to the data characteristics, t-tests and one-way ANOVA were used to compare the differences in the scores of different groups of subjects, to analyse the main factors affecting the stress levels of kidney transplant patients, and to perform multivariate linear regression analysis on the results with significant differences. *p* < 0.05 was used to indicate statistical significance.

### 2.9 Ethical considerations

The research protocol has been approved by the Ethics Committee of Xiang’an Hospital of Xiamen University (reference number XAHLL2025027). Informed consent was obtained from all participants before answering the questions. All data collected was anonymous and confidential to protect the privacy of participants.

## 3 Results

### 3.1 Basic information

A total of 202 valid questionnaires were collected in this study. The General demographic characteristics and disease-related information of kidney transplant patients are presented in Tables 1, 2.

**Table 1:** General demographic characteristics (*χ* ± s, *n* = 202).

Basic characteristics		Numbers *(N)*	Percentage (%)
Gender	Male	120	59.41
	Female	82	40.59
Age	18–30 years old	12	5.94
	31–45 years old	93	46.04
	46–60 years old	85	42.08
	Over 60 years old	12	5.94
Marital status	Married	163	80.69
	Unmarried/Other	39	19.31
Having children	Has children	160	79.21
	No children	42	20.79
Education level	Junior high school or below	65	32.18
	High school/Vocational school	68	33.66
	College/University or above	69	34.16
Employment status	Working/Individual business owner	119	58.91
	Unemployed/Retired	83	41.09
Religious beliefs	Yes	73	36.14
	No	129	63.86
From Min nan region	Yes	145	71.78
	No	57	28.22
Family monthly income	< 8,000 yuan	107	52.97
	8,000–15,000 yuan	65	32.18
	>15,000 yuan	30	14.85
Incurring debt	Yes	115	56.93
	No	87	43.07
Medical expenses as a proportion of family income	Less than 20%	55	27.23
	20–40% (including 20%)	74	36.63
	40–60% (including 40%)	44	21.78
	60–80% (including 60%)	18	8.91
	Over 80% (including 80%)	11	5.45
Main medical payment method	Urban Employee Basic Medical Insurance	151	74.75
	Urban and Rural Resident Basic Medical Insurance/Other	51	25.25
Lifestyle	Living with family	183	90.59
	Living with other relatives/Alone/Co - renting	19	9.41

**Table 2:** Disease-related conditions (χ ± s, *n* = 202).

Basic characteristics		Numbers *(N)*	Percentage (%)
Awareness of the primary disease	Clear	68	33.66
	Unclear	134	66.34
BMI	Normal	127	62.87
	Underweight/Overweight/Obese	75	37.13
Dialysis method before transplantation	Hemodialysis	154	76.24
	Peritoneal dialysis/Both	48	23.76
Duration of dialysis	Less than 1 year	68	33.66
	1–3 years (excluding 3 years)	72	35.64
	3–5 years (excluding 5 years)	40	19.80
	5 years or more	22	10.89
Waiting time for transplantation	Less than 1 year	95	47.03
	1–3 years (excluding 3 years)	84	41.58
	3 years or more	23	11.39
Post - operative time	Less than 1 year	20	9.90
	1–3 years (excluding 3 years)	23	11.39
	3–5 years (excluding 5 years)	68	33.66
	5 years or more	91	45.05
Post - operative complications	No complications	103	50.99
	1–2 types	78	38.61
	More than 3 types	21	10.40
History of infection	Yes	66	32.67
	No	136	67.33
Creatinine recovery status	Normal	108	53.47
	Abnormal	94	46.53
Presence of other diseases	Yes	112	55.45
	No	90	44.55
Awareness of the need to maintain a healthy lifestyle	Very clear	77	38.12
	Somewhat clear	91	45.05
	Generally clear	34	16.83

### 3.2 The stress levels of kidney transplant recipients

The overall mean stress score for kidney transplant patients was 57.78 ± 12.73, indicating an average stress level, with an average score of 2.41 ± 0.53 across all items. The average score for the economic situation dimension was the highest (2.91 ± 1.07), while the average score for the romantic relationship dimension was the lowest (1.86 ± 1.00), indicating that kidney transplant patients experience the most stress in terms of their economic situation, while experiencing relatively less stress in terms of their romantic relationships. The specific scores for each dimension are detailed in Table 3.

**Table 3:** Status of stress in kidney transplant patients (χ ± s, *n* = 202).

Dimension	Number of items	Total score	Average item score	Ranking of average score
Health impact	5	12.34 ± 3.31	2.47 ± 0.66	3
Romantic and marital psychology	2	3.73 ± 1.99	1.86 ± 1.00	5
Economic situation	5	14.53 ± 5.36	2.91 ± 1.07	1
Limitations in daily life	6	14.85 ± 4.70	2.48 ± 0.78	2
Support system	6	12.33 ± 4.54	2.05 ± 0.76	4
Total score	24	57.78 ± 12.73	2.41 ± 0.53	——

### 3.3 A single-factor analysis of the stress levels of kidney transplant recipients

The study findings indicate that factors such as household monthly income, debt status, awareness of the primary disease, waiting time for surgery, postoperative duration, postoperative complications, history of infection, serum creatinine recovery, presence of comorbidities, the proportion of medical expenses relative to household income, and knowledge of the necessity for long-term healthy lifestyle maintenance post-kidney transplantation significantly influence the stress levels of transplant recipients (*p* < 0.05), as shown in Table 4.

**Table 4:** An analysis of the factors influencing the stress levels of kidney transplant recipients.

Basic characteristics		Total score	*T/F*-value	*p*-value
Family monthly income	< 8,000 yuan	61.56 ± 13.33	11.362	0.000
	8,000–15,000 yuan	54.15 ± 9.87		
	>15,000 yuan	52.13 ± 11.99		
Incurring debt	Yes	62.70 ± 12.51	7.046	0.000
	No	51.26 ± 9.81		
Awareness of the primary disease	Clear	54.87 ± 10.22	0.020	0.020
	Unclear	59.25 ± 13.63		
Waiting time for transplantation	Less than 1 year	53.73 ± 12.20	5.357	0.005
	1–3 years (excluding 3 years)	58.07 ± 13.03		
	3 years or more	65.17 ± 11.40		
Post - operative time	Less than 1 year	52.50 ± 11.84	5.150	0.002
	1–3 years (excluding 3 years)	53.48 ± 13.01		
	3–5 years (excluding 5 years)	56.00 ± 12.73		
	5 years or more	61.65 ± 12.01		
Post - operative complications	No complications	53.15 ± 12.12	24.468	0.000
	1–2 types	60.31 ± 10.94		
	More than 3 types	71.10 ± 9.95		
History of infection	Yes	61.80 ± 11.79	3.201	0.002
	No	55.82 ± 12.76		
Creatinine recovery status	Normal	56.06 ± 12.10	−2.065	0.040
	Abnormal	59.74 ± 13.22		
Presence of other diseases	Yes	61.20 ± 12.12	4.45	0.00
	No	53.52 ± 12.25		
Medical expenses as a proportion of family income	Less than 20%	52.27 ± 10.57	9.70	0.00
	20–40% (including 20%)	58.22 ± 12.28		
	40–60% (including 40%)	58.89 ± 12.11		
	60–80% (including 60%)	64.67 ± 10.98		
	Over 80% (including 80%)	71.64 ± 13.57		
Awareness of the need to maintain a healthy lifestyle	Very clear	55.22 ± 15.08	7.619	0.001
	Somewhat clear	57.23 ± 10.53		
	Generally clear	65.03 ± 9.56		

### 3.4 Comparison of scores across different pressure levels

The results indicate that there are significant differences (*p* < 0.05) among groups with varying stress levels across five dimensions: health impact, psychological aspects of marriage and relationships, economic status, life restrictions, and support systems. As stress levels increase, kidney transplant patients experience a corresponding rise in perceived stress across all dimensions, as shown in Table 5.

**Table 5:** Comparing the dimension scores of different pressure level groups.

Dimension	Low- stress *(N = 62)*	Medium- stress *(N = 82)*	High- stress *(N = 58)*	*F*-value	*p*-value
Health impact	9.98 ± 2.78^b^	12.71 ± 2.80^a^	14.33 ± 3.01^ab^	35.876	0.000
Romantic and marital psychology	2.69 ± 1.21 ^b^	3.72 ± 1.74 ^a^	4.84 ± 2.39^ab^	20.931	0.000
Economic situation	10.23 ± 3.78 ^b^	14.24 ± 3.89 ^a^	19.55 ± 4.30^ab^	82.634	0.000
Limitations in daily life	10.84 ± 3.15 ^b^	14.93 ± 3.45 ^a^	19.03 ± 3.88^ab^	82.729	0.000
Support system	9.89 ± 3.53 ^b^	12.37 ± 4.20 ^a^	14.88 ± 4.58 ^ab^	21.943	0.000

a indicates *p* < 0.05 when compared with the low-stress group; b indicates *p* < 0.05 when compared with the medium-stress group.

### 3.5 Multifactorial analysis of stress status in kidney transplant recipients

A multivariate linear regression model was created using the stress score of kidney transplant patients as the dependent variable and incorporating variables with statistically significant results from the univariate analysis. The results indicate that factors such as debt status, clarity of the primary disease, waiting time for surgery, postoperative duration and complications, infection history, presence of comorbidities, proportion of medical expenses relative to household income and awareness of the need to maintain a healthy lifestyle in the long term after kidney transplantation are significant determinants of stress levels in kidney transplant patients (*p* < 0.05). These variables account for 48.5% of the total variance, as shown in Table 6.

**Table 6:** Results of the multiple linear regression analysis.

Dependent variable	Unstandardized		Standardized	*t*-value	*p*-value
	B	SE	Beta		
Constant	47.177	7.438		6.343	0.000
Family monthly income	−0.697	1.066	−0.04	−0.655	0.514
Incurring debt	−6.583	1.648	−0.257	−3.994	0.000
Awareness of the primary disease	4.599	1.446	0.171	3.18	0.002
Waiting time for transplantation	2.322	1.062	0.124	2.186	0.030
Post - operative time	1.81	0.784	0.138	2.309	0.022
Post - operative complications	4.193	1.13	0.221	3.71	0.000
History of infection	−3.993	1.471	−0.147	−2.715	0.007
Creatinine recovery status	0.223	1.397	0.009	0.16	0.873
Presence of other diseases	−3.448	1.495	−0.135	−2.306	0.022
Medical expenses as a proportion of family income	1.87	0.739	0.165	2.53	0.012
Awareness of the need to maintain a healthy lifestyle	2.45	0.966	0.137	2.537	0.012

*R* = 0.696, *R*^2=^0.485, *F* = 16.245, *p*<0.01.

## 4 Discussion

### 4.1 Financial burden

Financial burden is one of the main sources of stress for kidney transplant patients, affecting them throughout the treatment and recovery process. Studies have shown that high treatment costs and long-term financial stress directly affect patients’ mental health and quality of life and may negatively impact their recovery. Research by Wang et al. (34) revealed a significant correlation between the quality of life of kidney transplant recipients and per capita monthly family income, as well as the health insurance payment method (*p* < 0.05). This suggests that patients in poorer financial circumstances face greater difficulties during postoperative rehabilitation. Additionally, a high level of family support reduces the risk of glomerular filtration rate abnormality (OR = 0.278) and mitigates the negative prognostic impact of economic stress (36). Therefore, alleviating economic stress through social capital, such as family support and health insurance policies, is crucial for improving patients’ quality of life and rehabilitation outcomes.

For kidney transplant patients whose condition is relatively stable, helping them to improve their ability to adapt to work and maintain employment is important. Ma et al. point out that (37) finding a job again can reduce patients’ economic pressure, and that employment after transplantation is an important sign of recovery (38), as well as being a key part of overall well-being with important social implications. In theory, patients whose kidney function is normal 3 months after the transplant can return to work. However, most patients choose to wait until 6 months after the transplant before returning to work (39). Furthermore, research shows that kidney transplant patients are moderately prepared to return to work (40), but have a strong desire to do so. This shows that, despite having a strong desire to return to work, patients still face many concerns and obstacles in the employment process. This suggests that medical staff need to provide more psychological support during the perioperative period, and that society should provide more support and help, such as vocational training, employment guidance and the construction of barrier-free environments, to help patients better integrate back into society.

### 4.2 Disease-related situation

Research has shown that postoperative complications, infection history, delayed recovery of creatinine levels and comorbidities significantly increase patient stress, possibly due to health uncertainty and treatment complexity. Furthermore, higher stress levels during the waiting period for transplantation and in the early postoperative phase (short time after surgery) reflect preoperative anxiety and the psychological challenges of the postoperative adaptation period. This suggests that healthcare professionals need to strengthen perioperative psychological counseling and implement targeted interventions for high-risk patients.

High blood sugar levels are common in patients undergoing kidney transplantation, and are associated with surgical stress and the use of immunosuppressive agents (such as glucocorticoids and tacrolimus). The use of high doses of immunosuppressive agents and glucocorticoids after surgery inevitably causes metabolic disturbances, resulting in significant blood sugar fluctuations and difficulty in controlling blood sugar levels, as well as widespread tissue oedema (41). High blood sugar levels can exacerbate patients’ inflammatory responses and cardiovascular complications, and can further increase their psychological burden, leading to increased anxiety and depression. Therefore, in clinical management, it is important to monitor blood sugar levels closely and adjust the treatment plan accordingly to avoid significant blood sugar fluctuations, thereby alleviating patients’ psychological distress.

Drug adherence is an important part of managing kidney transplants. Patients need to take immunosuppressants long term, but some patients may not follow the medication plan strictly due to side effects of the drugs, cost or other reasons. Poor drug adherence increases the risk of rejection reactions and other complications (21), and can also lead to further psychological stress. Research shows that kidney transplant recipients’ adherence to immunosuppressant drugs needs to be improved, and that marital status and time since transplant are the main factors influencing adherence to medication (42). In clinical management, it is important to address patients’ medication adherence issues, providing personalised medication guidance and psychological support to help patients develop the right attitude toward medication and reduce their psychological burden.

### 4.3 Health awareness

Research has found that patients with an unclear understanding of the primary disease or who lack knowledge of postoperative health management have higher stress levels. This suggests that inadequate health education may limit patients’ sense of self-efficacy, further exacerbating their psychological burden. As the perception of disease worsens (43), patients’ fear of disease progression increases. Therefore, timely health education and public awareness campaigns are crucial in alleviating patients’ misperceptions of disease.

When a suitable kidney donor is found, the kidney transplant recipient will be urgently notified to come in for surgery, based on the matching criteria and their position on the waiting list. Due to the need to complete a series of preoperative preparations in a short time, the time available for preoperative health education is extremely limited. This time constraint makes it difficult for patients to fully understand the disease-related information and postoperative management points before surgery, thereby increasing postoperative anxiety due to information gaps.

Personalised interventions after kidney transplantation are particularly important. The “Knowledge, Attitude and Practice” video combined with the clinical pathway intervention model can significantly improve disease knowledge acquisition (*p* < 0.05), shorten the time to first get out of bed/eat after surgery, and reduce the 3-month readmission rate (44). Furthermore, Zhang et al. (45) found that a two-pronged intervention involving couples can improve medication adherence and reduce perceived burden (*p* < 0.001) and alleviated psychological distress. This family-involved approach not only enhances patients’ health behaviors, but also provides additional support through the family support system. Qualitative research has also highlighted the urgent need for health management and social reintegration information, and systematic education can alleviate the anxiety caused by uncertainty (46). Based on the health belief model (e.g., medication adherence interventions), strengthening patients’ sense of control over their disease can reduce medication concerns (47); Personalised health education can improve the electrolyte levels of kidney transplant patients, stabilize their vital signs, and enhance their health behaviors (48). Furthermore, electronic health literacy can promote the self-management abilities of kidney transplant patients (49). These studies demonstrate that multi-dimensional health education and personalised intervention strategies can effectively enhance patients’ health awareness, alleviate psychological distress, and improve post-operative recovery.

### 4.4 The multidimensionality and dynamic changes of pressure performance

Differences in the dimensions of health effects, life limitations, and support systems in the different stress level groups (*p* < 0.05) indicate that patients’ negative perceptions of physical and social functioning are enhanced across the board when stress is elevated. The age range of the participants was concentrated in the 30–60 age group. There were few participants in the over-60 age group, which made it difficult to reflect the pressure experienced by this group. Kidney transplantation requires a certain level of economic investment, and due to economic constraints, most older adult people often choose dialysis treatment. However, patients undergoing maintenance hemodialysis (MHD) are more likely to experience cognitive impairment, which is related to older age, lower educational attainment and higher HIF-1α levels (50). However, findings suggest (51) that overall survival and graft survival in older adults transplant recipients may be comparable to younger recipients. Compared with maintaining dialysis, patients over 65 years old who received a functional transplant experienced an increase in life years and quality-adjusted life years, indicating that they can benefit from kidney transplantation (52). However, older kidney transplant patients have a higher infection rate than younger patients, which may be related to age-related immunodeficiency (53). The pressure experienced by older adult patients who choose dialysis over kidney transplantation may be related to economic burden, treatment options, and concerns about quality of life in old age. This highlights the importance of promoting kidney transplantation among older aduls patients, while also addressing their economic and psychological needs.

It is worth noting that the relatively low level of psychological pressure on marriage in this observational study may be related to the fact that most of the samples are married and have children, and their marriage and childbearing situations have stabilized. This group is more concerned about survival needs, but still needs to pay attention to long-term psychosocial adaptation. This group is more concerned with survival needs, but long-term psychological and social adaptation still needs to be considered. The stability of marital relationships and the presence of a supportive family environment have a significant impact on the stress levels of kidney transplant patients, and adequate support can improve long-term transplant outcomes (13, 54).

Considering that the prevalence of chronic kidney disease is increasing year by year and gradually getting younger, more unmarried and childbearing young people may become patients in the future. A study by Marleen C. et al. (55) found that women of childbearing age after kidney transplantation have a strong desire to have children, but impaired graft function is something that many women experience after delivery, and the burden of disease and perception of risk are the determining factors for them not to continue their pregnancies. In addition, most pregnancies in women who become pregnant after transplantation are complicated by preeclampsia (56), which not only adds to the burden of childcare but also affects work energy. For women with kidney transplants (57), pregnancy moves them from “abnormal” to a state closer to that of a normal, healthy woman, but there is a need to be alert to the physical warnings that come with pregnancy. Men of childbearing age have the same needs for courtship, marriage, and childbearing, but some kidney transplant recipients do not have significantly improved fertility (58) and erectile dysfunction is prevalent, with recovery closely related to age (59). In addition, postoperative complications such as varicocele can cause patients’ testicular temperature to increase, leading to a decrease in sperm quality and thus affecting their fertility (60, 61)。.

For the multidimensionality and dynamic change characteristics of psychological stress, psychological intervention needs to integrate social support and cognitive reconstruction. On the one hand, social support plays an important role in alleviating psychological pain, and a study by Shu Lin et al. (62) at Xiang ya Third Hospital found that social support needs to indirectly reduce psychological pain by enhancing psychological resilience; on the other hand, guiding patients to express their concerns about transplantation and postoperative physical recovery can correct negative cognition and help cultivate positive emotions, thereby alleviating psychological stress (45). There are differences in the psychological mood of kidney transplant recipients at different treatment stages, with depression being more pronounced in the perioperative period, interpersonal sensitivity and anxiety being more pronounced in the follow-up period (63) In addition, a study by Li et al. (64) showed that positive cognitive mood contributed to psychological flexibility (*p* < 0.05), which further emphasized the importance of cognitive reconstruction in psychological interventions.

## 5 Conclusion

This study found that kidney transplant recipients exhibited moderate levels of stress, with economic pressure being the main source of stress for kidney transplant patients. Family economic status, disease-related factors and the extent to which patients understand post-operative rehabilitation knowledge significantly affect patients’ total stress scores. Based on these findings, the following targeted recommendations are proposed. First, provide economic aid to patients to reduce their financial burden and alleviate the psychological stress caused by financial issues. Second, support kidney transplant patients in returning to work, enabling them to earn an income and enhance their economic independence and psychological security. Third, strengthen the monitoring and management of post-operative symptoms, promptly addressing any complications to minimize the impact of disease-related factors on patients’ stress levels. Finally, conduct systematic post-operative rehabilitation education to improve patients’ understanding of the rehabilitation process and enhance their self-management abilities. In the future, multidisciplinary resources should be integrated to optimize comprehensive management and improve patients’ overall well-being and long-term prognosis.

### 6 Limitations and future research

Firstly, the data were derived from a single-centre sample, which may introduce selection bias. Validation across multi-centre, large-scale cohorts is necessary to confirm the generalizability of the findings. Secondly, a cross-sectional study can only reflect the pressure situation at a given time point and relies on subjective reports, which can lead to recall bias. However, subsequent longitudinal studies can be used to conduct forward-looking analytical research and reveal the dynamic changes in pressure. Thirdly, the model does not consider potential variables such as the quality of social support and coping mechanisms, which may limit its comprehensiveness. Future research should incorporate qualitative interviews to explore the complex psychosocial mechanisms underlying stress, and to inform the development of personalised intervention strategies.

## Data Availability

The raw data supporting the conclusions of this article will be made available by the authors, without undue reservation.
