# Correction: An analysis of the stress levels, influencing factors and countermeasures of kidney transplant recipients in a certain hospital

**DOI:** 10.3389/fpubh.2025.1731883

**Published:** 2025-11-06

**Authors:** Frontiers Production Office

**Affiliations:** Frontiers Media SA, Lausanne, Switzerland

**Keywords:** kidney transplantation, recipient, stress, influencing factors, countermeasures

Affiliations 1 and 2 were incorrectly ordered, affecting author superscript numbering. It was erroneously given as:

Jingyi Jiang^1†^, Lin Wu^1†^, Leiqun Xiong^2†^, Bin Zhao^1*^, Shaoping Tian^1^, Houzhao Wang^1*^ and Xiaoying Lv^2*^

^1^Department of Clinical Laboratory, Xiang'an Hospital of Xiamen University, School of Public Health, Xiamen University, Xiamen, Fujian, China

^2^Department of Transfusion, Zhongshan Hospital (Xiamen), Fudan University, Xiamen Clinical Research Center for Cancer Therapy, Xiamen, Fujian, China

The correct affiliation numbering and order is:

Jingyi Jiang^2†^, Lin Wu^2†^, LeiqunXiong^1†^, Bin Zhao^2^, Shaoping Tian^2^, Houzhao Wang^2*^ and Xiaoying Lv^1*^

^1^Department of Transfusion, Zhongshan Hospital (Xiamen), Fudan University, Xiamen Clinical Research Center for Cancer Therapy, Xiamen, Fujian, China

^2^Department of Clinical Laboratory, Xiang'an Hospital of Xiamen University, School of Public Health, Xiamen University, Xiamen, Fujian, China

The original version of this article has been updated.

